# Hydrocarbon Degradation and Enzyme Activities of *Aspergillus oryzae* and *Mucor irregularis* Isolated from Nigerian Crude Oil-Polluted Sites

**DOI:** 10.3390/microorganisms8121912

**Published:** 2020-11-30

**Authors:** Michael Dare Asemoloye, Solveig Tosi, Chiara Daccò, Xiao Wang, Shihan Xu, Mario Andrea Marchisio, Wenyuan Gao, Segun Gbolagade Jonathan, Lorenzo Pecoraro

**Affiliations:** 1 School of Pharmaceutical Science and Technology, Tianjin University, 92 Weijin Road, Nankai District, Tianjin 300072, China; asemoloyemike@gmail.com (M.D.A.); wang_xiao1996@163.com (X.W.); xsh1007@126.com (S.X.); pharmgao@tju.edu.cn (W.G.); 2 Laboratory of Mycology, Department of Earth and Environmental Sciences, University of Pavia, Via S. Epifanio 14, 27100 Pavia, Italy; solveig.tosi@unipv.it (S.T.); chiara.dacco01@universitadipavia.it (C.D.); 3 Mycology & Applied Microbiology Group, Department of Botany, University of Ibadan, Ibadan 200284, Oyo State, Nigeria; Gbolagadejonathan@gmail.com

**Keywords:** pollutants, dose inhibition response, extracellular enzymes, fungi, hydrocarbon degradation, tolerance, bio-treatment

## Abstract

Many free-living saprobic fungi are nature recruited organisms for the degradation of wastes, ranging from lignocellulose biomass to organic/inorganic chemicals, aided by their production of enzymes. In this study, fungal strains were isolated from contaminated crude-oil fields in Nigeria. The dominant fungi were selected from each site and identified as *Aspergillus oryzae* and *Mucor irregularis* based on morphological and molecular characterization, with site percentage incidences of 56.67% and 66.70%, respectively. Selected strains response/tolerance to complex hydrocarbon (used engine oil) was studied by growing them on Bushnell Haas (BH) mineral agar supplemented with the hydrocarbon at different concentrations, i.e., 5%, 10%, 15%, and 20%, with a control having dextrose. Hydrocarbon degradation potentials of these fungi were confirmed in BH broth culture filtrates pre-supplemented with 1% engine oil after 15 days of incubation using GC/MS. In addition, the presence of putative enzymes, laccase (Lac), manganese peroxidase (MnP), and lignin peroxidase (LiP) was confirmed in culture filtrates using appropriate substrates. The analyzed fungi grew in hydrocarbon supplemented medium with no other carbon source and exhibited 39.40% and 45.85% dose inhibition response (DIR) respectively at 20% hydrocarbon concentration. An enzyme activity test revealed that these two fungi produced more Lac than MnP and LiP. It was also observed through the GC/MS analyses that while *A. oryzae* acted on all hydrocarbon components in the used engine oil, *M. irregularis* only degraded the long-chain hydrocarbons and BTEX. This study confirms that *A. oryzae* and *M. irregularis* have the potential to be exploited in the bio-treatment and removal of hydrocarbons from polluted soils.

## 1. Introduction

An increase in anthropogenic activities has affected the natural environment in many ways. Studies have been reported on the occurrence of several pollutants and their various products in different environments [1,2,3]. In particular, the extraction, refinement, and transportation of petroleum oil and products are of critical concerns throughout the world in terms of environmental contamination [4,5,6]. Over 1.5 billion tons of petroleum oil is transported yearly, and despite prevention strategies, some significant amount still spills into the environment due to either operational issues or accidents. Petroleum hydrocarbons are generally characterized by many carbon bonds, which could bind to other compounds to form exceptional multi-complex structures, such as aliphatic alkanes/alkenes, chlorinated hydrocarbons or polycyclic aromatic hydrocarbons (PAHs), potentially harmful to the environment [7,8]. 

Many hydrocarbon complexes can alter the soil or water conditions in nature. They can percolate through the ecosystems and accumulate both in animal and plant tissues, thus exerting various toxic effects including cancer induction, mutations, and malfunctioning of respiratory and central nervous systems [9,10,11,12,13]. For this reason, PAHs and some organic compounds like mono-aromatic hydrocarbons have been listed as priority pollutants by the United State Environmental Protection Agency [14,15]. In addition, hydrocarbon contaminants in soil have been shown to affect the microbial communities negatively; gasoline for example have been shown to exert poisonous effect on different soil microorganisms [16]. Many other petroleum-based products like engine oil, diesel, or paraffin are non- or semi-soluble in water, causing significant water contamination and less bioavailability for microbial degradation [17]. Moreover, used engine oil contains several mixtures of hydrocarbons, engine additives and toxic metals like Al, Cr, Cu, Fe, Ni, Si, and Pb [18].

Remediation of soils polluted with hydrocarbons has attracted much attention over the last few decades. A variety of methods such as chemical/electrokinetic separation, photo-oxidation, extraction/thermal treatments, and soil flushing have been suggested to salvage the environment from the impact of petroleum spill and hydrocarbon contamination [19,20,21]. However, bioremediation is becoming a more attractive among other strategies used for pollution containment, being non-evasive, low cost, and environment-friendly [22,23,24]. More specifically, microbial degradation, among other available bioremediation technologies, has been adopted mainly for treatment of hydrocarbon impacted soils [25,26,27]. Many filamentous fungi have been reported as bioremediation agents due to their verse mycelial networks and enzyme secreting activities. Most rot fungi produce high redox potential enzymes such as manganese peroxidase (MnP), laccases (Lac), and lignin peroxidases (LiP) for the oxidation of lignin. These enzymes are not generally substrate-specific as they can oxidize a wide range of xenobiotics, including pesticides, plastics, and hydrocarbons [26,27]. 

A continuous search for naturally existing microbes with bioremediation potentials, especially for most concerned pollutants like the persistent organics, has been performed in the last few decades. Mycelial degradation mechanisms on many complex hydrocarbon pollutants have been clearly described in a large number of studies [17,18,25,27]. In this work, culturable filamentous fungal strains were isolated from two crude oil impacted sites in Niger Delta area, Nigeria. It was hypothesized that fungi inhabiting crude oil contaminated soils over a long time could have developed the ability to degrade/mineralize the hydrocarbons due to their survival in such environment. Dominant strains from the investigated sites were characterized for enzyme secreting activities and hydrocarbon degradation.

## 2. Materials and Methods

### 2.1. Study Sites and Sampling

Two sites that have been frequently exposed to oil spillage in Niger Delta area of Nigeria were selected for this study. These investigated sites included Yorla-10 (latitude 4°394534′ N and longitude 7°261123′ E) and Effurun (latitude 5°344926″ N, longitude 5°465611″ E) communities as shown in Map Supplementary File Figure S1. Yorla-10 (Yorla oil field 10 location) is included in a cluster of oil wells at Kwawa community located in Khana Local Government Area of Rivers State, while Effurun community is located in Uvwie Local Government Area of Delta State, Nigeria.

Soil samples (100 g each) were collected from 30 sampling points on each site at a depth of 5–20 cm from soil surfaces. The samples were kept in sterile cryovial tubes and brought to laboratory for fungal isolation. Physicochemical properties of these polluted soil samples were analyzed using standard analytical procedures [28] as presented in the supplementary file (Tables S1 and S2). Used engine oil was obtained from a private Italian company in this study and treated as complex hydrocarbon mixture, this mixture consisted of short and long-chain aliphatics/aromatics as well as polycyclic aromatic hydrocarbons [7]. The composition of this oil was defined using Thermo Scientific DSQII single-quadrupole gas chromatography coupled with a mass spectrophotometry (GC/MS) system.

### 2.2. Fungal Strains

Fungal strains were isolated from each soil sample in Petri dishes, on sterile potato dextrose agar (PDA) medium supplemented with ampicillin (100 mg/L) to suppress bacteria interference. The soil samples were subjected to serial dilution at 10^−6^ using sterile water, inoculated on prepared PDA, and then incubated at 30 °C, in the darkness, for three days to allow fungal growth. Pure cultures were made from primary PDA plates by carefully picking and inoculating each colony into a new plate. Isolated strains were grouped based on plate morphology, while the number of samples from which a particular strain was isolated was used to calculate its percentage incidence in that site [29,30,31].

$$\mathrm{Percentage}\;\mathrm{Incidence}=\;\frac{\mathrm{Number}\;\mathrm{of}\;\mathrm{samples}\;\mathrm{having}\;a\;\mathrm{particular}\;\mathrm{fungus}}{\mathrm{Total}\;\mathrm{number}\;\mathrm{of}\;\mathrm{samples}\;\mathrm{analyzed}\;}\;\times \;100$$

The fungal strain having the highest incidence was regarded as the dominant strain and was selected from each site for further studies.

### 2.3. Morphology and Molecular Identification of Dominant Strains

The living fungal cultures of selected dominant fungi were deposited in the Laboratory of Mycology, at the Department of Earth and Environmental Sciences, University of Pavia, Italy and LP Culture Collection (personal culture collection held in the laboratory of Prof. Lorenzo Pecoraro), at the School of Pharmaceutical Science and Technology, Tianjin University, Tianjin, China. The strains were grown on different media, including Czapek yeast extract agar (CYA), Czapek Dox solution agar (CZA), Malt extract agar (MEA), and Potato dextrose agar (PDA), with incubation temperature of 25 °C. The selected strains were characterized based on morphological and molecular methods. For morphological characterization, conidial/mycelial color, reverse plate color, colony diameter, seriation, vesicle, presence/absence of cleistothecial wall, presence/absence of Hulle cells and sclerotia, and zygospore/ascospore/conidia structures were observed, following keys for morphological identification [32]. 

Molecular identification of the selected strains was made using PCR amplification of the internal transcribed spacer (ITS) region. Fungal genomic DNA was extracted using the hexadecyl trimethyl ammonium bromide (cTAB) extraction buffer made up of 50 mM Tris Buffer pH 8.0, 100 mM EDTA, 150 mM NaCl, and 1% mercaptoethanol. Fungal mycelia were transferred into 2.0 mL Eppendorf tubes (400 mg each) and stored at 80 °C overnight, then kept in ice for 15 min to allow cell thaw. The genomic DNA extraction was carried out as described by Möller et al. [33], modified by Asemoloye et al. [23,24]. DNA concentration was checked using a nanodrop spectrophotometer (Thermo Fisher Scientific, Waltham, MA, USA) at light absorbance of 260 and 280 nm respectively, while DNA quality was checked in 1% agarose gel electrophoresis. 

The PCR ITS amplification was carried out using the universal primer pair pITS4-F (5′-TCCGTAGGTGAACCTGCCG-3′) and pITS1-R (5′-TCCTCCGCTTATTGATATGC-3′) according to White et al. [34]. 2 µL of genomic DNA (100–500 ng) was added to the PCR mixtures containing 25 µL T5 Tsingke colony PCR master mix, 1.5 µL of each primer (5 µg), and 20 µL of deionized water. The thermal cycler was set at PCR condition of 98 °C/3 min initial denaturation step followed by 35 cycles of 98 °C/10 s, annealing temperature of 55 °C/10 s, extension temperature of 72 °C/15 s followed by final extension temperature of 72 °C for 2 min. Products obtained were checked on gel electrophoresis, purified using QIAquick PCR purification kit (Qiagen) following the manufacturers prescriptions and sent to Genewiz, Tianjin, China for sequencing.

Sequences were edited to remove vector sequences and to ensure correct orientation and assembled using Sequencher 4.1 for MacOsX (Genes Codes, Ann Arbor, MI, USA). Sequence analysis was conducted with BLAST searches against the National Center for Biotechnology Information (NCBI) sequence database (GenBank; http://www.ncbi.nlm.nih.gov/BLAST/index.html) to determine the closest sequence matches that enabled taxonomic identification. Fungal DNA sequences amplified from strains (B-Yorla10 and C-Effurun) isolated from oil-contaminated soil in Nigeria were submitted to GenBank under accessions MW114835–MW114836. DNA Sequences were aligned using Clustal X 2.1 [35]. Phylogenetic analysis was performed using MEGA 7.0 [36], and neighbour-joining tree was constructed by Kimura 2-parameter distances, with bootstrapping of 1000 replicates. *Umbelopsis nana* was used as outgroup to root the *Mucor* tree, while the *Aspergillus* fungi tree was rooted with *Aspergillus peyronelii* [37,38,39,40].

### 2.4. Hydrocarbon Tolerance Test

The ability of selected fungi to respond and tolerate the used engine oil was studied by growing them on BH agar, a synthetic growth medium containing only minerals without any carbon source. Composition of this medium included magnesium sulphate (0.20 g/L), calcium chloride (0.20 g/L), monopotassium phosphate (1.0 g/L), dipotassium phosphate (1.0 g/L), ammonium nitrate (1.0 g/L), ferric chloride (0.05 g/L) at pH 7.0 ± 0.2. BH agar medium was supplemented with used engine oil as the only carbon source at 5, 10, 15 and 20% concentration, while a plate amended with dextrose (10 g/L) was used as control. The selected fungal strains were inoculated on 90 × 15 mm Petri plates containing the mixture mentioned above in six replications, while their hydrocarbon tolerance capacity was studied through mycelial growth measurements every 24 h for eight days. Data obtained were used to calculate fungal dose inhibition response (DIR) to hydrocarbons [41,42].

$$DIR=\;\frac{\mathrm{Growth}\;\mathrm{rate}\;\mathrm{on}\;\mathrm{hydrocarbon}\;\mathrm{plate}}{\mathrm{Growth}\;\mathrm{rate}\;\mathrm{on}\;\mathrm{control}\;\mathrm{plate}}\;\times \;100$$

### 2.5. Enzyme Activities of the Selected Dominant Fungi

The analysed enzymes included lignin peroxidase (LiP) manganese peroxide (MnP), and laccase (Lac), which were selected due to their largely recognized redox-potential activities on a wide range of hydrocarbon compounds. The latter enzymes belong to the natural ligninolytic oxidative system that has been reported in many fungi associated with degradation of several pollutants [27,43,44,45]. Colorimetric enzyme assay was performed by growing the fungi on Petri plates containing 15 mL of sterile malt extract agar (MEA) medium. MEA was prepared in three groups; a group with no chemical substrate, another group was supplemented with 0.05% guaiacol and 1 mM CuSO_4_ as enzyme substrates while the last group was supplemented with 0.5 mM 2,2-Azino-bis-3- benzthiazoline-6-sulfonic acid (ABTS) and 1 mM CuSO_4_ [46,47]. 

A pilot enzyme activity study was conducted to characterize enzymes that are secreted by these fungi; fungal inoculum was prepared by cutting out a mycelial disc (50 mm diameter) from the outer edge of an actively growing fungus and transferred aseptically into a glass tube containing 10 mL sterile water with broken glasses. The mixture was vortex for 1 min and kept at −4 °C until further use. Bushnell Haas (BH) broth was prepared according to manufacturer’s prescription (Sigma Aldrich, St. Louis, MO, USA) and supplemented with 0 (control), 2.5, and 5% of used engine oil in conical flasks. The flasks were tightly corked and sterilized at 121 °C. 1 mL of the mycelium-water mixture was transferred into different BH flasks with corresponding engine oil concentrations and kept in a shaker incubator at 120 rpm, 30 °C. Aliquots of 300 µL were taken from each flask for enzyme activity tests after 3, 12, and 24 days of incubation. This experiment was later repeated by adjusting the oil concentrations to 0.5%, 1.0%, and 1.5% of the BH medium with control (no oil supplemented). This time the enzyme activity tests were carried out at day 3, 6, and 9 days of incubation. 

Activities of secreted LiP in each culture aliquot were studied based on the evolution of hydrogen peroxide in the presence of veratryl alcohol and sodium tartrate serving as substrates [43,44,45]. For this analysis, a reaction volume containing 300 µL veratryl alcohol (2 mM), 300 µL sodium tartrate (100 mM at pH 4.5), and 40 µL H_2_O_2_ (0.4 mM) was mixed with 300 µL of the culture aliquots. Absorbance was measured at 460 nm (A460) in a 10 min interval using a UV visible spectrophotometer (Perkin Elmer LAMDA 25). LiP activities was calculated and reported in U/mL. Manganese-dependent peroxidase activity was determined through the evolution of hydrogen peroxide in the presence of magnesium sulphate and sodium tartrate as substrates, while phenol served as an indicator to monitor the reaction changes with time. Specifically, 40 µL magnesium sulphate (1 mM), 300 µL sodium tartrate (100 mM at pH 4.5), 0.01% phenol and H_2_O_2_ (60 µL) were mixed for reaction with the culture extracts (300 µL) according to the protocol followed by Paszcymski et al. [47] and Ameen et al. [44]. The decline in absorbance measured at 460 nm (A460) in a 10 min interval, and MnP activity was calculated and recorded in U/mL. In addition, Lac activity was determined according to the method of Novotny et al. [48] and Juhasz et al. [49] by measuring the oxidation of ABTS. 300 µL of sodium tartrate (100 mM at pH 4.5), and 300 µL ABTS (1 mM) were mixed with 300 µL of the culture extracts, and the decline in absorbance was measured at 490 nm (A490) in 5 min period intervals at 30 °C. Lac activity was calculated and recorded in U/mL (1 enzyme unit (U) = 1 μmol/min, where μmol refers to the amount of substrate converted) (https://www.physiologyweb.com).

### 2.6. Hydrocarbon Degradation Analysis

The ability of selected fungal strains to degrade used engine oil was studied in BH broth. 20 mL of this medium was supplemented with the used engine oil at 1% concentration (v/v) in conical flasks and inoculated with each fungus, while flasks with no fungus inoculated was treated as control. Mycelial plugs (5 mm diameter) were taken from 4-day old pure fungal cultures and transferred into the 20 mL BH-Hydrocarbon mixture kept in a shaker incubator at 80 rpm and 30 °C. The control was analyzed for hydrocarbon content using GC-MS at time (T_0_) and compared with medium treated with each fungus at day-15 of incubation. To determine the amount of hydrocarbon degraded, 1 mL aliquot solution was drawn from each treatment and analyzed for hydrocarbon content at the inoculum moment and after 15 days of incubation. The 1 mL aliquot solution took was dissolved in 1 mL of dichloromethane (CH_2_Cl_2_), and vortexed vigorously for 1 min and then left on rack for 5 min to let the organic layer separate. The organic layer was carefully pipetted out into another tube, centrifuged for 3 min at 3000 rpm and then analyzed by gas chromatography coupled with mass spectrophotometry (Thermos Scientific DSQII single-quadrupole GC/MS system, Austin, TX, USA). 

The GC/MS conditions were in split mode with injection at 250 °C, oven temperature was 70 °C for 1 min, 70–120 °C at 5 °C/min, 120–260 °C at 8 °C/min, and held at 260 °C for 5 min. A Restek Rxi-5Sil MS 30 m × 0.25 mm × 0.25 μm film thickness capillary column was used with helium as the carrier gas at a constant flow rate of 1.0 mL/min. The transfer line temperature was 270 °C and the ion source temperature was 250 °C. Electron ionization mode was used with 70 eV, and the ions were registered in full scan mode in a mass range of *m/z* 35–800 amu. Chromatogram acquisition, detection of mass spectral peaks, and waveform processing were performed using Xcalibur MS Software Version 2.1 (Thermo Scientific Inc., Waltham, MA, USA). The assignment of chemical structures to chromatographic peaks was done based on comparison with the databases for the GC/MS National Institute of Standard and Technology (NIST) Mass Spectral Library (NIST 08) and Wiley Registry of Mass Spectral Data (8th Edition). The percentage content of each component was directly computed from the peak areas in the GC/MS chromatogram.

## 3. Results

### 3.1. Identification of Fungi Isolated from Crude Oil Polluted Sites

Chemical analyses for the two aged crude oil polluted soils used for fungal isolation in this study confirmed elevated concentration of hydrocarbons and toxic metals as presented in Tables S1 and S2. Fourteen fungal strains were isolated from polluted soils and subjected to percentage incidence test to determine the dominant strains (Table 1). In the first polluted site (Yorla-10), fungal strain B appeared in 20 out of 30 samples, thus being the dominant fungus with 66.67% incidence. The second crude oil polluted site (Effurun) on the other hand had strain C as the dominant fungus, appearing in 17 out of 30 samples collected, with 56.67% incidence.

The response and tolerance of these dominant fungi to complex hydrocarbon mixture (used engine oil) is presented in Table 2. It was observed that the two selected fungi survived the hydrocarbon mixture as high as 20% concentration in growth medium. Their ability to tolerate hydrocarbon presence was calculated based on dose inhibition response (DIR). Strain B had DIR of 75.52, 59.96, 51.24 and, 45.85 DIR on medium supplemented with 5, 10, 15, and 20% hydrocarbon respectively, while strain C had DIR of 53.97, 46.69, 39.07, and 39.40 respectively. The studied fungi were identified, based on morphological and molecular methods, as *Mucor irregularis* (strain B-Yorla10) and *Aspergillus oryzae* (C-Effurun).

**Figure 1:**
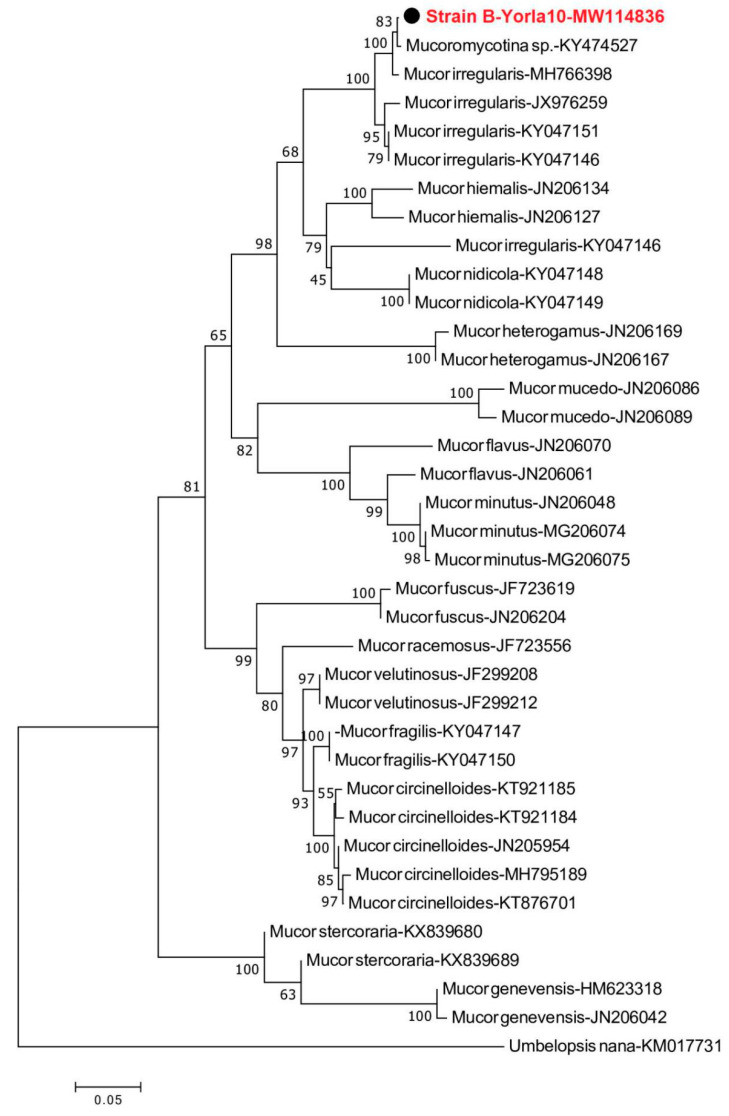
Neighbour-joining phylogenetic tree showing the relationship between the *Mucor* sequence from strain B-Yorla10 isolated from oil-contaminated soil in this study and selected database relatives. Kimura 2-parameter distances were used. Bootstrap values are based on percentages of 1000 replicates. The tree was rooted with *Umbelopsis nana* as outgroup.

**Figure 2:**
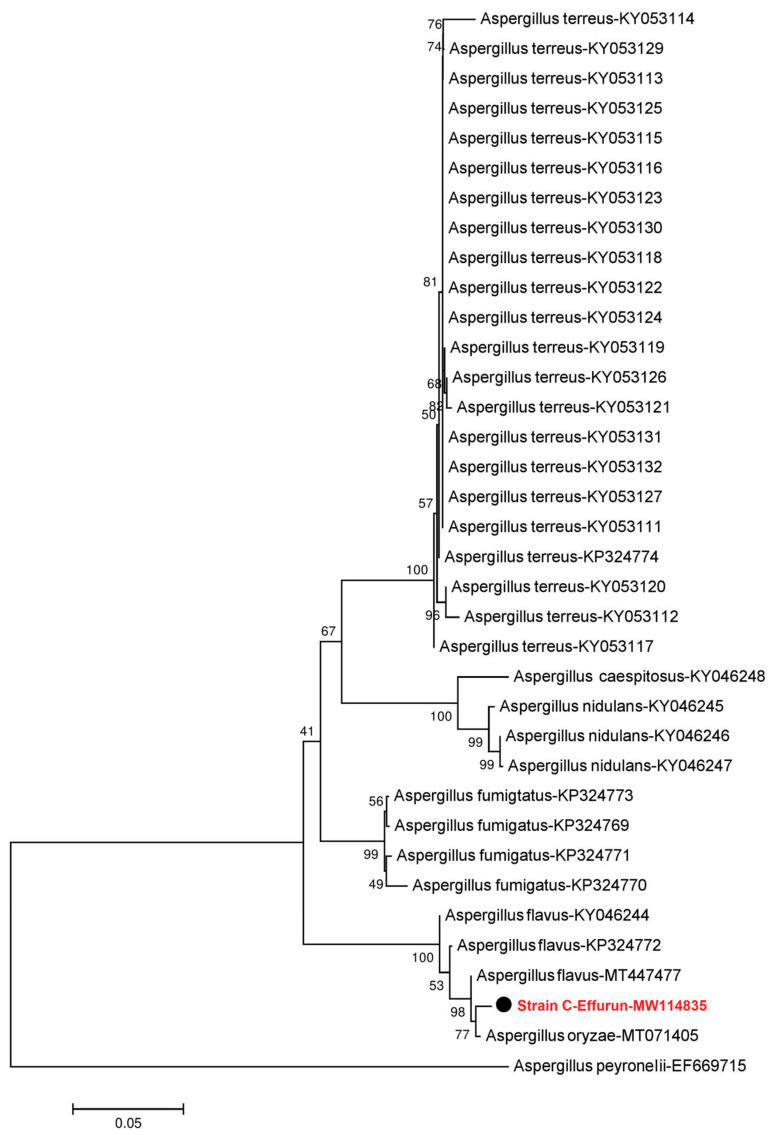
Neighbour-joining phylogenetic tree showing the relationship between the *Aspergillus* sequence from strain C-Effurun isolated from oil-contaminated soil in this study and selected database relatives. Kimura 2-parameter distances were used. Bootstrap values were based on percentages of 1000 replicates. *Aspergillus peyronelii* (EF669715.1) was used as the outgroup.

Phylogenetic analysis clarified the relationships of fungi isolated from crude oil-contaminated soil within *Mucor* and *Aspergillus*. Sequences retrieved from the Nigerian sites analyzed strains could be aligned with sequences from fungi isolated from a variety of soils, animal faeces, water and oil-contaminated surface soils [37,38,39]. The neighbour-joining tree of *Mucor* fungi revealed that the sequence obtained from isolated *Mucor* strain B-Yorla10 in this study was closely related to KY474527-*Mucoromycotina* sp. previously found in the roots of *Drynaria quercifolia* in Philippines and *Mucor irregularis* found from rice (Figure 1). The phylogenetic tree from the *Aspergillus* dataset showed that the sequence amplified from strain C-Effurun clustered into a single well-supported clade (Figure 2) including endophytic fungi (MT071405) previously found in the kernels of *Coix lachrymal-jobi* cultivars (the best BLAST match, with 99% identity) and rhizosphere soil of *Lycium barbarum* in China.

### 3.2. Assay and Activities of Enzymes Secreted by Selected Fungal Strains

**Figure 3:**
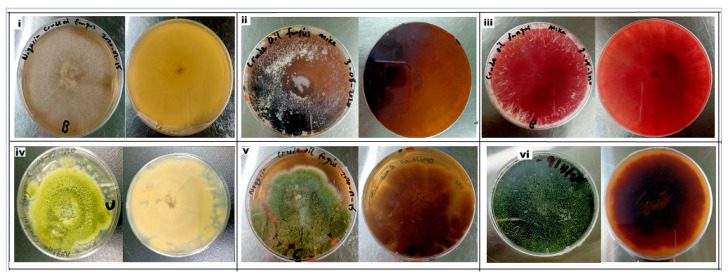
Plate assay for colorimetric screening of ligninolytic enzymes produced by the isolated fungi. (**i**) Control—*Mucor irregularis* strain B-Yorla10 growing on MEA without chemicals (**ii**) *Mucor irregularis* strain B-Yorla10 showing reddish brown coloration on reaction with MEA supplemented with CuSO4 and Guaiacol (**iii**) *Mucor irregularis* strain B-Yorla10 showing reddish brown coloration on reaction with MEA supplemented with CuSO4 and ABTS (**iv**) Control—*Aspergillus oryzae* strain C-Effurun growing on MEA without chemicals (**v**) *Aspergillus oryzae* strain C-Effurun showing reddish brown coloration on reaction with MEA supplemented with CuSO4 and Guaiacol (**vi**) *Aspergillus oryzae* strain C-Effurun showing reddish brown coloration on reaction with MEA supplemented with CuSO4 and ABTS.

**Figure 4:**
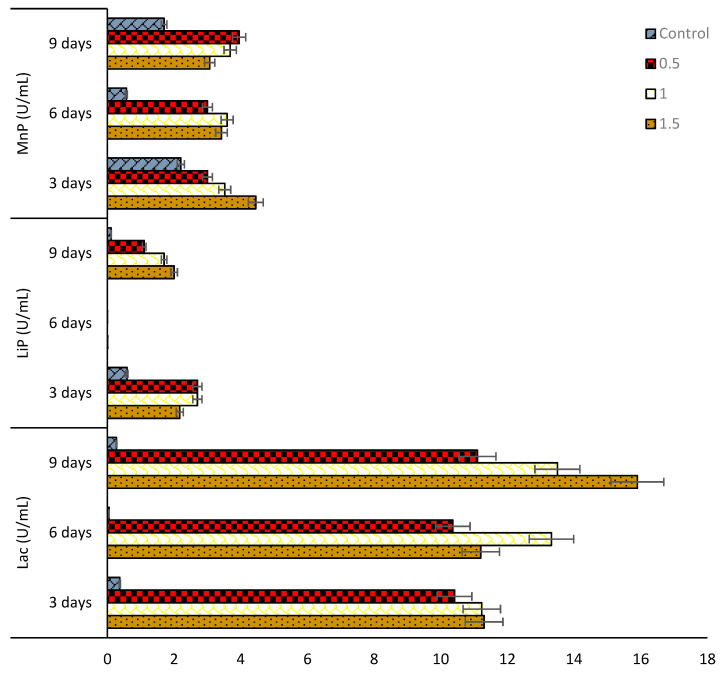
Activities of extracellular enzymes produced by *Mucor irregularis* B-Yorla10 isolated from a crude oil polluted site in Nigeria.

**Figure 5:**
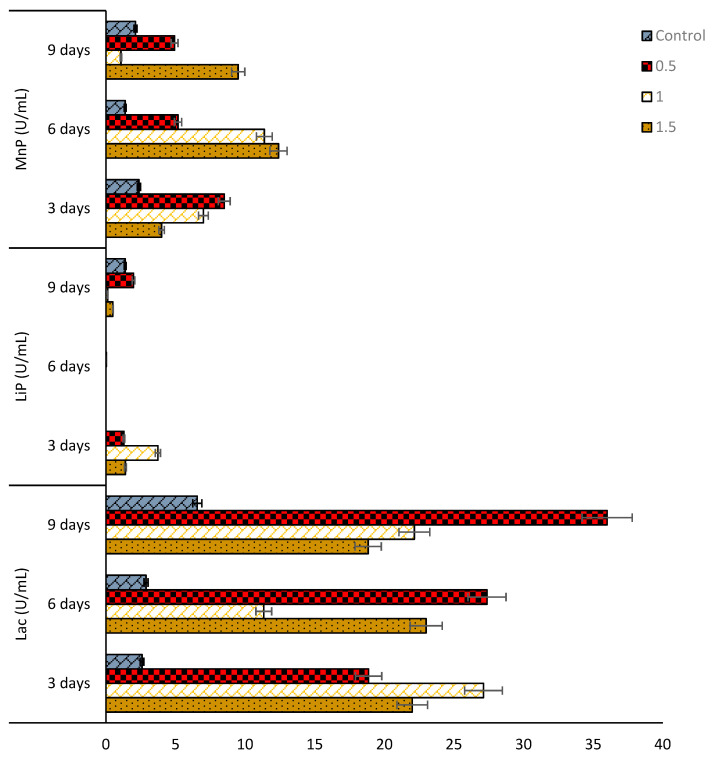
Activities of extracellular enzymes produced by *Aspergillus oryzae* C-Effurun isolated from a crude oil polluted site in Nigeria.

In this study, it was demonstrated that the two isolated fungal strains, *Mucor irregularis* (B-Yorla10) and *Aspergillus oryzae* (C-Effurun) possess the ability to produce ligninolytic enzymes (LiP, MnP, and Lac). The plate assay showed enzyme secretions by the two strains. Indeed, the analyzed mycelia formed significant reddish-brown color on plates supplemented with different substrates as compared to control (Figure 3). Results from preliminary enzyme activity test for these strains is presented in supplementary Table S3. Generally, the two fungi showed enzyme activities in medium not supplemented with oil (control) but in low amount (Figure 4 and Figure 5). It was also observed that the studied strains produced enzymes in response to oil concentrations in their growth medium and, in any case, they both showed more production of Lac as compared to LiP and MnP as presented in Figure 4 and Figure 5. *M. irregularis* (B-Yorla10) at day 13 of incubation showed highest Lac activities of 15.00, 13.90, 11.07 U/mL in BH medium supplemented with 1.5%, 1.0%, and 0.5% engine oil respectively (Figure 4). *A. oryzae* (C-Effurun) showed highest Lac activities of 36.0 and 27.37 U/mL at day-9 and day-6 respectively, while its least Lac activity of 2.11 U/mL was recorded on day-3. Strain C-Effurun showed highest MnP activities of 12.40 and 11.38 U/mL in medium supplemented with 1.5 and 1.0% oil respectively (Figure 5). Both fungi showed some peroxidase activities; strain B-Yorla10 showed highest expression of 2.70 U/mL of LiP in medium supplemented with 0.5 and 1% oil respectively at day-3, while strain C-Effurun showed highest expression of 3.73 U/mL of LiP in medium supplemented with 1% oil also at day-3 (Figure 4 and Figure 5). Generally, both strains grew in BH medium with no oil supplement (control), but they showed better enzyme activity in media supplemented with oil as the only carbon source. 

### 3.3. Hydrocarbon Degradation

**Figure 6:**
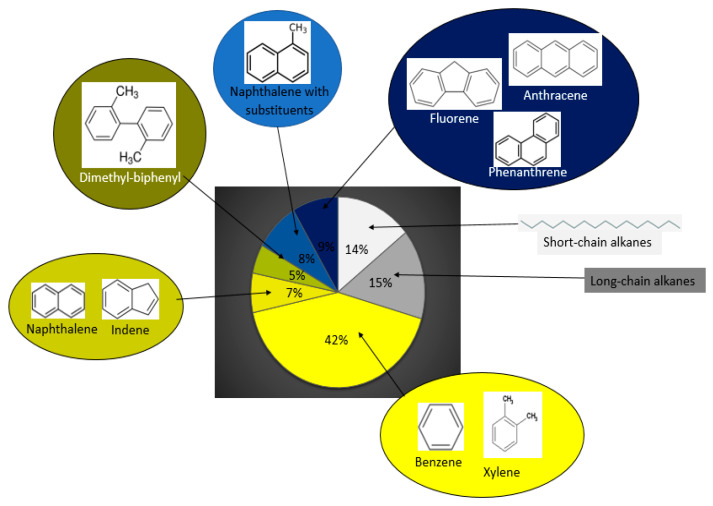
Composition of the used engine oil as revealed through GC/MS analysis.

**Figure 7:**
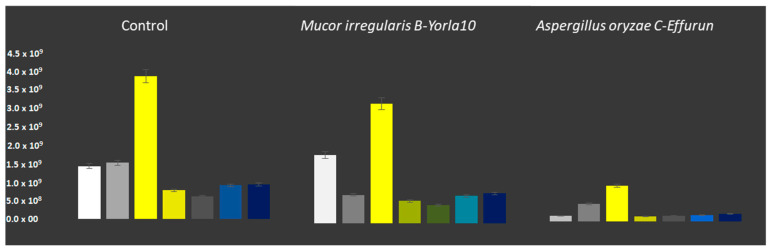
Degradation of hydrocarbon fractions in used engine oil by selected filamentous fungi. Degradation of used engine oil by *Mucor irregularis* and *Aspergillus oryzae*. The compounds were grouped in classes of Benzene (yellow), Naphthalene and Indene (dark-yellow), Dimethyl-biphenyl (brown), Naphthalene and substituents (blue), the group of Anthracene, Fluorene and Phenanthrene (dark-blue), Short-chain alkanes (white) and Long chain alkanes (grey).

The composition of engine oil used in this study was analyzed by GC/MS (Figure 6). The oil included aliphatic components (e.g., C_1_–C_20_ and C_20_–C_50_), aromatics (e.g., benzene, toluene, ethylbenzene, and xylene isomers (BTEX), Alkyl benzenes, Alkyl indenes, Alkyl tetralines, Alkyl biphenyls, polycyclic aromatics (PAHs)), and other compounds, such as methyl esters. The most abundant compounds in the used engine oil were the BTEX, C_20_–C_50_ aliphatics, and PAHs together making 67% of the total mixture (Figure 6). It was observed that both fungi modified the hydrocarbon composition of the used engine oil in terms of as presented in Figure 7. The chromatogram of oil composition for the control and treatments is presented in Supplementary File Figure S2. It was observed after a 15-day incubation that *A. oryzae* (C-Effurun) significantly reduced several components of the used engine oil as compared to *Mucor irregularis* B-Yorla10. More specifically, *A. oryzae* (C-Effurun) reduced all the hydrocarbons in the used engine oil, while the strain *Mucor irregularis* B-Yorla10 only showed a significant degradation activity of long-chain alkanes and BTEX. However, the degradation of long-chain alkanes by the latter fungus resulted in increased short chain alkanes in the treatment as compared to the control.

## 4. Discussion

In this study, the two most frequently occurring fungi *Aspergillus oryzae* and *Mucor irregularis* were isolated from aged crude oil-polluted sites in Niger Delta Area of Nigeria. The isolated *Mucor* strain (B-Yorla10) was highly phylogenetically close to *Mucoromycotina* sp. discovered by Aban et al. [50,51] as a symbiotic fungus from the root of epiphytic fern, *Drynaria quercifolia* in Philippines being the dominant species with high colonization rate [50]. In another study performed later by the same authors, this fungal strain isolated from fern showed significant capability in protecting rice seedlings against drought [51]. *M. irregularis* found from rice by Cai. W. in an unpublished study also revealed a close relationship with our isolated *Mucor* strain. This result proves the wide ecological distribution of *Mucor* in soil and as plant associate, and may indicate the potentiality of *Mucor* to be applied in the field of environmental protection and agricultural industry. Chukwura et al. reported hydrocarbon degrading potentials of *M. racemosus* isolated from soil samples collected from a mechanic shop at Awka, Anambra State, Nigeria [52]. Our result represents a new record of the hydrocarbon degrading potential of *M. irregularis* isolated from oil-polluted soils in Nigeria. 

The result of phylogenetic analysis showed that strain C-Effurun was similar to *Aspergillus oryzae* isolated as endophytic fungus from kernels of *Coix lachrymal-jobi*. cultivars by Li et al. in China [53]. The latter study demonstrated that *A. oryzae* could produce a variety of enzymes including proteinases, amylases, glycases, cellulases and phytases, which promoted the degradation of the unabsorbable crude fiber and phytin [53]. The high enzyme-producing activity of *A. oryzae* was confirmed in our study where the analysed strain showed capabilities to produce laccase (Lac), manganese peroxidase (MnP), and lignin peroxidase (LiP). These results enriched our knowledge on the enzyme resource pool secreted by *A. oryzae*, suggesting that studied fungus could be regarded as a potential candidate for exploitation in microbial chemistry and hydrocarbon degradation.

In this study, it was found that both fungi could tolerate varying concentrations of used engine oil and utilize hydrocarbons as sole carbon source. While *M. irregularis* grew faster on solid BH medium supplemented with 20% used engine oil, *A. oryzae* tend to grow slower. On the contrary, the hydrocarbon degradation results showed that *A. oryzae* had more degradative potential as compared to *M. irregularis* which was confirmed by its higher enzymes production. Our findings confirm that fungal tolerance to complex hydrocarbons is not a function of their degradation ability, and also that the enzyme secreting ability in fungi enhance hydrocarbon degradation faster than tolerance [23,25]. Several previous studies have shown the degradative capabilities of *Aspergillus* and *Mucor* species [54,55,56]. For example, Barnes et al. [57] reported 12 *Aspergillus* species able to degrade crude oil and derivatives, including *Aspergillus flavus, A. sydowii,* and *A. versicolor*. According to Harms et al. [58] and Banerjee et al. [59], many fungal species such as *Aspergillus niger*, *A. flavus*, and *Rhizomucor variabilis* can transform complex hydrocarbons to nontoxic compounds through different catabolic pathways. This is also in accordance with our observation that there was an increase in short-chain aliphatic hydrocarbons in medium used to grow *M. irregularis* as compared to their initial concentration in the used engine oil. This study showed that the investigated crude oil polluted sites were colonized by fungi having adapted mechanisms to degrade and mineralize hydrocarbons as sole carbon source. Our results are in accordance with a previous research where *A. terreus* isolated from hydrocarbon oil contaminated mangrove sediments from Red Sea coast of Saudi Arabia was found to accumulate significantly higher biomass, produce extracellular enzymes, and degrade 28–56% hydrocarbons [60]. 

Some previous studies carried out on degradation of specific PAHs by *Aspergillus* fungi showed significant degradation of anthracene and naphthalene [61]. *A. terreus* isolated from a polycyclic aromatic hydrocarbons polluted soil degraded and metabolized Pyrene and benzo(a)pyrene [62,63,64], while *A. niger* was used for bioremediation of anthracene by AI-Jawhari [65]. In the latter work, degradation rate of benzo[a]pyrene in co-culture of *A. niger* and *Penicillium funiculosum* medium doubles that of medium without *A. niger*. AI-Jawhari [66] found 95% maximum crude oil bioremediation by mixed culture of *A. niger* and *A. fumigatus* after 28 days. Mahmoud et al. [67] found that *A. terreus* isolated from kerosene-polluted soil had a potential for both lipase production and crude oil degradation. The authors also suggested the use of hydrocarbon-polluted soils for fungal isolation in further studies seeking for highly lipase-producing and crude oil-removing fungi [68].

Although this study demonstrated that *A. oryzae* degraded more hydrocarbons than *M. irregularis*, the latter fungal species was found to act more specifically on long-chain hydrocarbons, thus resulting in increased short chain hydrocarbon concentrations after 15-day incubation as compared to the initial concentration in the used engine oil. In a previous study, *Mucor racemosus* was found to degrade about 50% of benzo[a]pyrene when subjected to metabolism evaluation using HPLC-DAD-MS technique [69,70]. Fungi in the *Mucoromycotina* have been continuously listed along with *Ascomycota* and *Basidiomycota* as major fungal *taxa* that are commonly involved in the biodegradation of oil [56,71,72,73,74,75,76]. *Mucor mucedo* was specifically characterized for exopolymeric substances produced during PAH degradation process in a work performed by Jia et al. [77]. Srinivasan and Viraraghavan [78,79] confirmed the application of *Mucor rouxii* and *Absidia coerulea* for biomaterial production in chitosan and walnut shell media for the removal of oil from aqueous solutions. 

Enzymes produced by many filamentous fungi have been demonstrated to aid the degradation/mineralization of hydrocarbons. For instance, biodegradation of diesel oil hydrocarbons was enhanced with enzyme preparations from *M. circinelloides* [80]. Biodegradation of slop oil by endophytic *Bacillus cereus* EN18 was enhanced by adding lipase from *Rhizomucor miehei* [81,82]. Asemoloye et al. [23,24] found the presence of laccase and peroxidase genes in *A. niger* and correlated their expressions with hydrocarbon degradation of Nigeria Bonny light crude oil. Similarly, it was earlier reported by Ramirez et al. [83] that laccases and peroxidases were involved in fungal environmental response to different concentrations of carbon, nitrogen and other pollutants, such as toxic metal ions, pesticides, and hydrocarbons. In the latter study, the authors explained that these fungal enzymes also serve as an abiotic stress signal in temperature shock, various lengths of day light, and increased xenobiotic pollution [84]. 

Laccase and peroxidase enzymes are produced by fungi as secondary metabolites. Therefore, these enzymes production may be affected by available carbon or nitrogen concentrations in the substrates where the fungi grow [85,86,87]. This is in agreement with our observation that the concentration and chemical nature of engine oil supplemented in culture medium as the only carbon source significantly affected the fungal growth and their enzyme secretion capacity. It was also observed that the two analyzed fungi produced more laccase than peroxidases. Laccase is a multi-copper phenol enzyme that has been reported to take different roles in oxidation of several phenolic, aromatic amine compounds. This enzyme acts by oxidizing several phenolic and aromatic compounds, using oxygen as terminal electron acceptors, as reported in different fungal species [87]. Laccase is becoming more popular in biotreatment of different recalcitrant materials and waste effluents from many industries. It has been well reported for many applications like the removal of aromatic phenolic compounds, deoxigenation of many hydrocarbon oils, treatment of waste waters, dye removals, and pulp bio-bleaching. 

## 5. Conclusions

In this study, the dominant fungal strains isolated from two crude oil polluted sites were identified as *Aspergillus oryzae* and *Mucor irregularis*. The two fungi showed high tolerance to varying concentrations of a complex hydrocarbon mixture (used engine oil) and demonstrated hydrocarbon degradation abilities. The different enzyme expressions and activities showed by the analyzed fungi could be helpful for their survival in contaminated environments, by allowing them to utilize the hydrocarbons present in the substrate as nutrients. Therefore, these two strains are potential candidates for the remediation of hydrocarbon polluted soils. However, further studies are needed to understand, from genetic and biochemical points of view, the hydrocarbon degradation mechanisms of these fungi, and therefore enhance their degradation performance. This can be achieved through the use of the traditional recombinant DNA technology and advanced gene manipulation tools such as the CRIPR-Cas systems [88]. Efforts are ongoing in our laboratory to obtain more information on the chemical processes and pathways used by these fungi in degrading hydrocarbons. We are also aiming to characterize possible biosynthetic gene clusters/genes associated with laccase expression in these two fungi and attempt to further enhance their expressions in heterologous yeast hosts. 

## Figures and Tables

**Table 1:** Incidence and dominant fungal strains isolated from crude oil polluted sites.

Strain Code	B-Yorla10		C-Effurun	
	Incidence (N = 30)	% Incidence	Incidence (N = 30)	% Incidence
A	9	30	1	3.33
B	20	66.67	13	43.33
C	13	43.33	17	56.67
D	0	0.00	0	0.00
E	5	16.67	11	36.67
F	3	10.00	2	6.67
G	0	0.00	3	13.33
H	11	36.67	0	0.00
I	1	3.33	1	3.33
J	5	16.66	11	36.67
K	2	6.67	3	10.00
N1	9	30.00	0	0.00
N2	6	20.00	5	16.67
N3	11	36.67	0	0.00

**Table 2:** Hydrocarbon tolerance and dose inhibition by two selected fungal strains from crude oil polluted sites.

Fungal Strain	Oil Concentration	Growth Rate (cm/day)	DIR (%)
B	0%	4.82	100.00
B	5%	3.64	75.52
B	10%	2.89	59.96
B	15%	2.47	51.24
B	20%	2.21	45.85
C	0%	3.02	100.00
C	5%	1.63	53.97
C	10%	1.41	46.69
C	15%	1.18	39.07
C	20%	1.19	39.40

## Supplementary Materials

The following are available online at https://www.mdpi.com/2076-2607/8/12/1912/s1. Table S1: Chemical properties of the contaminated soils. Table S2: Toxic metal composition of the contaminated soils. Table S3: Preliminary enzyme activities of *Aspergillus oryzae* and *Mucor irregularis* isolated from Nigerian crude oil polluted sites. Figure S1: Location of crude oil polluted sites from which fungi were isolated Figure S2: Degradation of complex hydrocarbon mixture (engine oil) by selected filamentous fungi as determined by GC/MS analysis.
