# Supplementary material for: Hydrocarbon Degradation and Enzyme Activities of Aspergillus oryzae and Mucor irregularis Isolated from Nigerian Crude Oil-Polluted Sites
Source: Microorganisms. 2020 Nov 30;8(12):1912. doi: 10.3390/microorganisms8121912 (PMC7761101; doi:10.3390/microorganisms8121912)
Supplement: Supplementary file 1 [file microorganisms-08-01912-s001.pdf]

Table S1. Chemical properties of the contaminated soils.

Soil	pH (H_2O)	THC (mg kg^{-1})	TOC (%)	Phosphate ($mgkg^{-1}$)	Nitrate (mg kg^{-1})	Conductivity (μS cm^{-1})	Nutrient (mg/kg)				
							Ca	Mg	Na	K	P
**Yorla10**	4.80	582.90	4.12	77.45	141.45	7.23	3.33	0.00	0.00	31.66	20.63
**Effurun**	5.20	572.20	4.8	73.56	129.99	6.37	3.20	2.11	0.01	31.70	14.67

Table S2. Heavy metal composition of the contaminated soils.

Soil	Heavy metals (mg/kg)									
	Cd	Cu	Pb	Fe	Ni	Cr	Hg	Co	As	Zn
**Yorla 10**	7.32	15.39	41.44	98.21	2.65	4.60	68.11	16.10	54.1	16.45
**Effurun**	5.76	18.80	44.31	45.00	2.65	2.00	52.00	17.23	39.00	9.00

Table S3. Preliminary enzyme activities of *Aspergillus oryzae* and *Mucor irregularis* isolated from Nigerian crude oil polluted sites.

Day of Incubation	Hydrocarbon conc.	Strain	Lcc (OD/min)	LiP (OD/min)	MnP (OD/min)
Day 7	0%	B-Yorla10	0.05	-	0.18
		C-Effurum	-	-	-
	2.50%	B-Yorla10	0.07	-	0.91
		C-Effurum	0.05	0.01	-
	5%	B-Yorla10	0.09	0.05	0.78
		C-Effurum	0.05	-	-
Day 14	0%	B-Yorla10	0.01	-	0.12
		C-Effurum	0.07	-	0.01
	2.50%	B-Yorla10	0.17	-	0.12
		C-Effurum	0.10	-	-
	5%	B-Yorla10	0.09	-	0.12
		C-Effurum	0.08	-	0.03
Day 21	0%	B-Yorla10	0.09	0.07	0.18
		C-Effurum	-	0.01	-
	2.50%	B-Yorla10	0.10	0.02	0.95
		C-Effurum	0.10	-	0.05
	5%	B-Yorla10	0.06	0.06	0.92
		C-Effurum	0.09	-	0.08

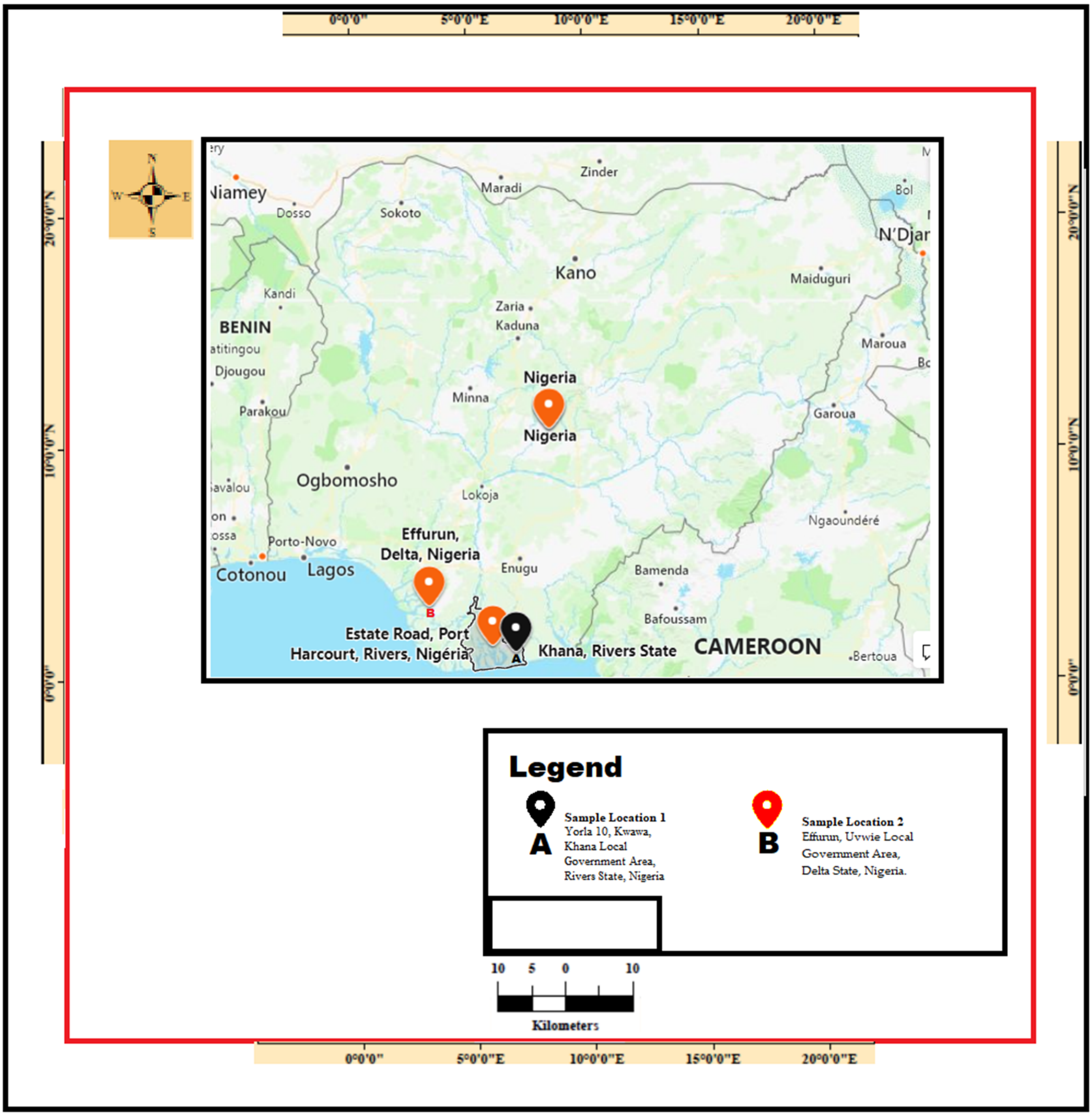

Figure S1. Location of crude oil polluted sites from which fungi were isolated.

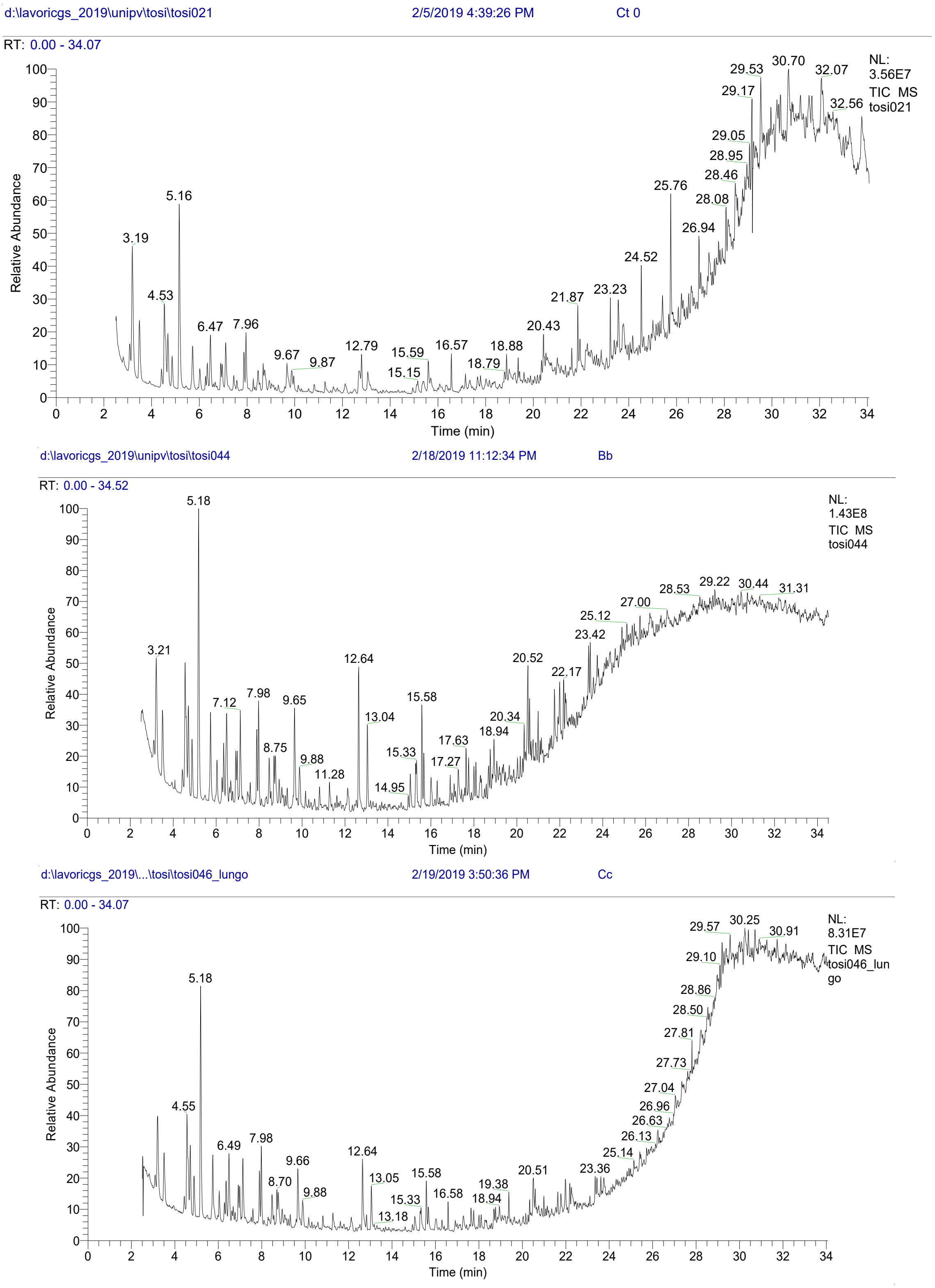

Figure S2. Degradation of complex hydrocarbon mixture (engine oil) by selected filamentous fungi as determined by GC/MS analysis.
